# EMS providers do not use FOAM for education

**DOI:** 10.1186/s12245-018-0189-4

**Published:** 2018-05-24

**Authors:** Joshua Bucher, Colleen Donovan, Jonathan McCoy

**Affiliations:** Department of Emergency Medicine, Rutgers – Robert Wood Johnson Medical School, 1 RWJ Place, MEB 104, New Brunswick, NJ 0890 USA

**Keywords:** FOAM, Social media, Education, FOAMed, EMS, Emergency medical services

## Abstract

**Background:**

Free open access to medical education (FOAM, #FOAM) is the free availability of educational materials on various medicine topics. We hope to evaluate the use of social media and FOAM by emergency medical services (EMS) providers.

**Methods:**

We designed an online survey distributed to EMS providers with questions about demographics and social media/FOAM use by providers. The survey was sent to the American College of Emergency Physicians (ACEP) EMS Listserv of medical directors and was asked to be distributed to their respective agencies. The survey was designed to inquire about the providers’ knowledge of FOAM and social media and their use of the above for EMS education.

**Results:**

There were 169 respondents out of a total of 523 providers yielding a response rate of 32.3%. Fifty-three percent of respondents are paramedics, 37% are EMT-Basic trained, and the remainder (16%) were “other.”

The minority (20%) of respondents had heard of FOAM. However, 54% of respondents had heard of “free medical education online” regarding pertinent topics. Of the total respondents who used social media for education, 31% used Facebook and 23% used blogs and podcasts as resources for online education.

Only 4% of respondents stated they produced FOAM content. Seventy-six percent of respondents said they were “interested” or “very interested” in using FOAM for medical education. If FOAM provided continuing medical education (CME), 83% of respondents would be interested in using it.

**Conclusion:**

Social media is not used frequently by EMS providers for the purposes of FOAM. There is interest within EMS providers to use FOAM for education, even if CME was not provided. FOAM can provide a novel area of education for EMS.

## Background

Free open access to medical education (FOAM, #FOAM) is the free availability of educational materials on various medicine topics. It is composed of various resources, including blog posts, podcasts, articles, short essays, reviews, videos, and other forms of educational material. The use of FOAM has increased significantly in popularity in the last decade of emergency medicine education [1]. Residents and faculty are using FOAM on a regular basis, and research has found that the use of social media increases their review of primary literature [2]. Residency programs are utilizing various FOAM resources in their didactic schedule in lieu of traditional lecture-based learning. Furthermore, the Council of Emergency Medicine Residency Directors (CORD) has released a position statement and guidelines on the use of social media and FOAM for residency education due to its importance [3].

Although the reasons for the use of social media in emergency medicine residents and faculty vary, there is widespread acceptance of social media in emergency medicine for communication as well as for the utilization of FOAM [4]. However, there is no scientific data on the use of social media or FOAM in emergency medical services (EMS). EMS and emergency medicine treat patients in a continuum of care; therefore, it is reasonable to assume that FOAM would be useful for EMS as well as emergency medicine.

In our study, we hope to evaluate the use of social media and FOAM by EMS providers. Hopefully, this study raises awareness of the availability of FOAM and its use by EMS providers for education.

## Methods

We designed an online survey distributed to EMS providers with questions about demographics and social media/FOAM use by providers. The survey was sent to the American College of Emergency Physicians (ACEP) EMS Listserv of medical directors and was asked to be distributed to their respective agencies. The medical directors who distributed the study were asked to email the corresponding author with the size of their organization so a response rate could be calculated. The respondents likely represent a convenience sample of EMS providers.

The survey was designed to inquire about the providers’ knowledge of FOAM and social media and their use of the above for EMS education. The survey was screened by several EMS healthcare providers prior to generalized distribution to assess for appropriateness. The survey was conducted using SurveyMonkey, a commercially available survey website and tool. Descriptive statistics were extracted from SurveyMonkey and reviewed prior to manuscript preparation.

Our study received approval from the Institutional Review Board at Rutgers University – Rutgers Biomedical and Health Sciences IRB.

## Results

**Fig. 1:**
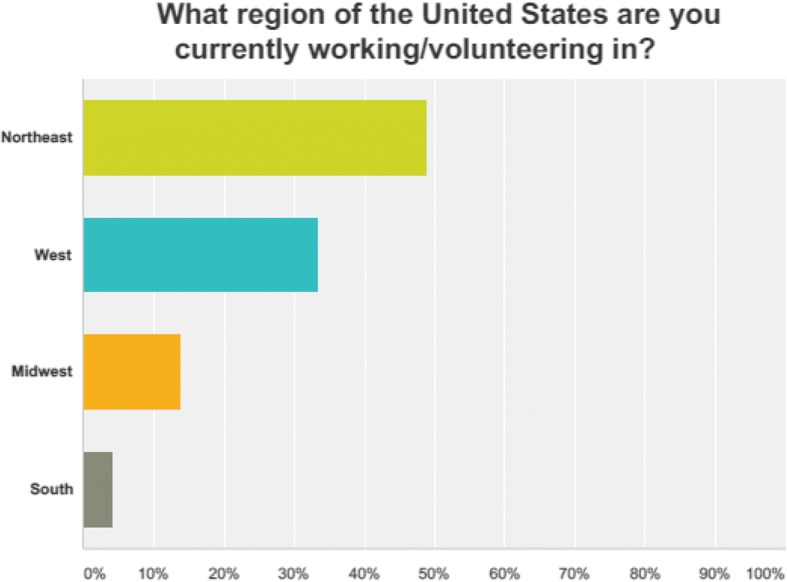
Regional distribution of respondents

**Fig. 2:**
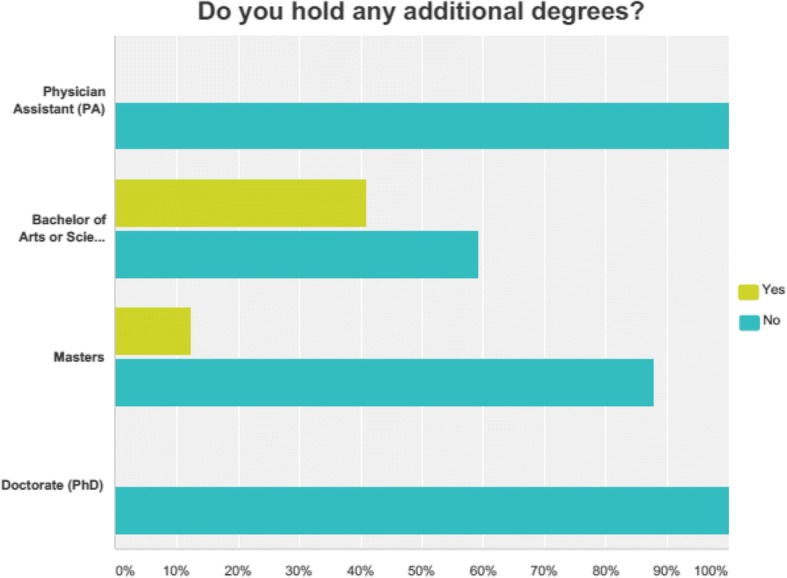
Additional degrees

**Fig. 3:**
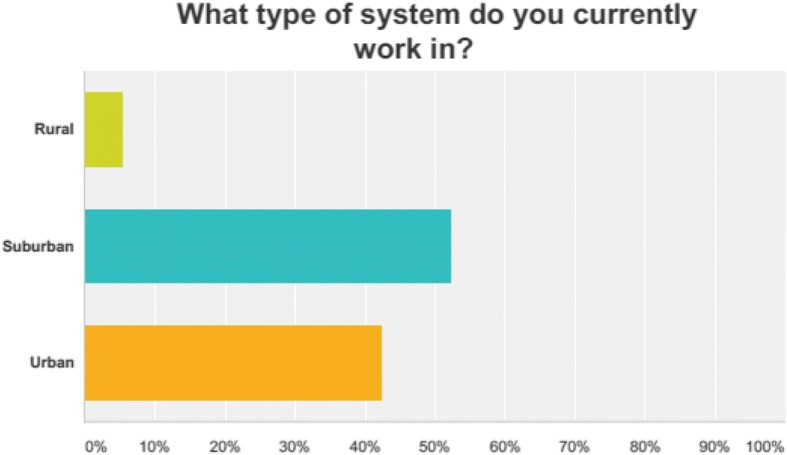
Type of system of respondents

There were 169 respondents out of a total of 523 providers yielding a response rate of 32.3%. Eighty-one percent of the respondents were male. The mean age of respondents was 39 years with an average of 17 years of experience per provider. Fifty-three percent of the respondents are paramedics, 37% are EMT-Basic trained, and the remainder (16%) are distributed between the remaining options. Eighty-one percent were involved in paid EMS. The majority (52%) practice in a suburban environment. Please refer to Figs. 1, 2, and 3 for further demographic information.

The minority (20.4%) of respondents had heard of the specific phrase, FOAM. However, 53.6% of respondents had heard of “free medical education online about topics that relate to EMS.” Of the total respondents, 30.7% used Facebook, 23.0% used blogs, and 23.5% podcasts as resources for online education. 72.6% reported using Facebook for “reasons other than medical education.” Only 8.0% of patients had reported using Twitter for FOAM.

Table 1 shows how frequently FOAM was used by the respondents for medical education.

**Table 1:** Rates of using FOAM resources

Resource	Yes	No	Total # respondents
Twitter	7.98% (13)	92.02% (150)	163
Facebook	30.72% (51)	69.28% (115)	166
Blogs	23.03% (38)	76.97% (127)	165
Podcasts	23.49% (39)	76.51% (127)	166

**Fig. 4:**
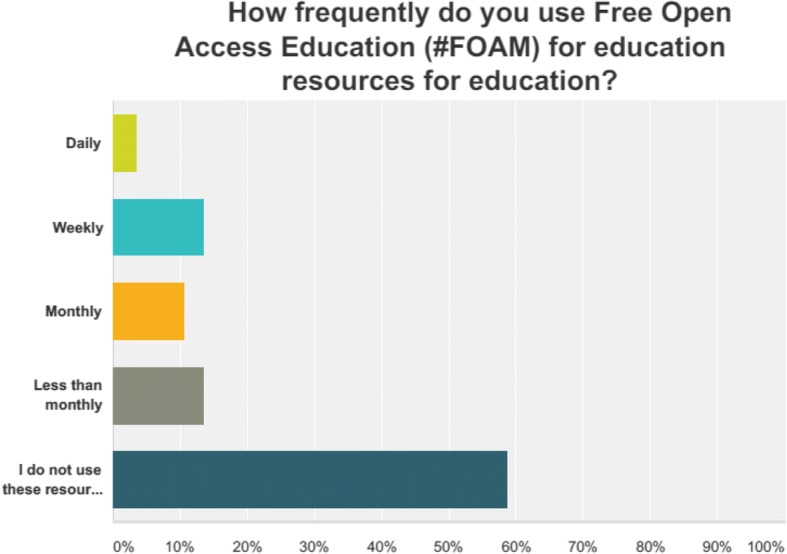
Frequency of use

Only 4.1% of respondents stated they produced FOAM content. 76.3% of respondents said they were “interested” or “very interested” in using FOAM for medical education. If FOAM provided CME, 84.5% of respondents would be interested in using it (Fig. 4).

The Appendix includes a selected group of responses to the open-ended question of, “what FOAM resources do you use?”

## Discussion

In the current era of increasing use of FOAM resources for medical education, the unfamiliarity of this resource by EMS providers is alarming. These are easily accessible, free sources of information on many topics that pertain to EMS. There are many FOAM resources that are now peer-reviewed and have been recommended in the medical literature [5, 6]. Furthermore, there have been two peer-review processes put forth by separate groups to identify quality FOAM resources [5, 6]. Likewise, there are FOAM resources that are dedicated to topics relevant to the practice of EMS, which can be a huge area for continuing education and improvement.

Eighty percent of our respondents were not aware of the phrase “free open access medical education” or the acronym “FOAM.” This is concerning, given the amount of attention FOAM has received in the emergency medicine community.

CORD released an expert consensus opinion regarding the use of social media for resident education. They promote the “adoption of social media as a valuable graduate medical education tool” for residency education [3]. This use of social media could be extrapolated into EMS, especially into the training of new paramedics.

Paramedic programs could incorporate the use of social media for many topics. FOAM could be incorporated in initial training of patients for EKG interpretation, critically injured patients, and airway management, in addition to others. FOAM also has a continuing education component; physician continuing medical education (CME) credit can be obtained from a variety of sources. FOAM resources, combined with an appropriate assessment, could be utilized by EMS for continuing education credit for EMS providers.

Likewise, continuing education could benefit from FOAM. FOAM resources could be utilized to provide current literature reviews, updates in guidelines from the professional organizations, and current practice trends. The utilization of FOAM and asynchronous learning would be a novel method of paramedic education.

## Limitations

There were several limitations to discuss. The study was a survey that was sent out and is self-reported, which leads to a significant amount of bias.

The response rate was disappointing. We were hoping for a response rate above 75% based on the recent publications and interest in FOAM in emergency medicine.

The small number of responses, overall, limits the generalizability of our data. In addition, nearly half of all patients were from the northeast, which may skew the results.

Most respondents were male. This may lead to a smaller representation of female prehospital provider’s knowledge on the topic.

## Conclusion

EMS providers have little familiarity with FOAM resources. FOAM resources have significantly increased in popularity in emergency medicine in the last few years and have tremendous potential to improve EMS education. Future novel EMS educational efforts should focus on the incorporation of FOAM resources.

## Appendix

### “Please list any specific Free Open Education Access (#FOAM) resources that you use.” 37 total answers

EMS1.com

JEMS.com

iTunes

PulmCCM

EMCrit

LITFL

RogueMedic

Resus ME

St. Emlyns

Flightbridge

ERCast

“Nursing podcasts”

Reddit

YouTube

EMS12Lead.com

http://hqmeded-ecg.blogspot.com/

SMACC

Prehospitalmed.com

## Abbreviations

ACEP: American College of Emergency Physicians
CME: Continuing medical education
CORD: Committee of residency directors
EMS: Emergency medical services
EMT: Emergency medical technician
FOAM: Free open access to medical education
